# Lumen-Apposing Metal Stents in the Management of Complex Pelvic Abscesses

**DOI:** 10.3390/diagnostics14242854

**Published:** 2024-12-18

**Authors:** Kenneth W. Chow, Nicholas A. Cumpian, Ranjit Makar, Pejman Zargar, Fouzia Oza, Idrees Suliman, Viktor Eysselein, Sofiya Reicher

**Affiliations:** 1 Department of Medicine, Harbor-UCLA Medical Center, Torrance, CA 90502, USA; 2 Division of Gastroenterology, Harbor-UCLA Medical Center, Torrance, CA 90502, USA

**Keywords:** LAMS, pelvic, abscess, EUS, drainage

## Abstract

Background: Lumen-apposing metal stents (LAMS) are utilized in a wide range of therapeutic gastrointestinal applications. We present our experience with LAMS-assisted drainage of complex pelvic abscesses at a large safety-net hospital. Methods: EUS-guided LAMS placements for pelvic abscesses from July 2020 to June 2024 were analyzed. Data were collected on patient demographics, procedure indications, fluid collection size, stent characteristics, stent dwell time, and complications. All cases underwent multidisciplinary review with Surgery and Interventional Radiology (IR) prior to LAMS-assisted drainage; all were deemed not amenable to drainage by IR. Results: Eleven patients underwent EUS-guided drainage of complex pelvic abscesses with cautery-enhanced LAMS. Diverticulitis was the most common cause of abscesses (n = 6; 55%). The average time from presentation to drainage was 7 days (1–18). The average abscess size was 7.2 cm (3.9–12.0 cm). The most common LAMS size was 15 mm × 10 mm; each was placed through the left colon and rectum with both technical and clinical success. All abscesses completely resolved with a mean stent dwell time of 28 days (17–42 days). After stent removal, the fistula was not routinely closed. No complications such as stent migration, bleeding, or perforation occurred. There were no recurrences and no patients required additional surgical or IR procedures with a mean follow-up of 25 weeks (SD 35.6). Conclusions: Adequate drainage is the cornerstone of pelvic abscess management, but IR or surgical access can be challenging, with inadequate drainage and prolonged hospitalization leading to significant morbidity. In our experience, EUS-guided, LAMS-assisted drainage provides a safe and effective alternative for managing pelvic abscesses.

## 1. Introduction

Pelvic abscesses are recognized complications of gastrointestinal and genitourinary tract infections such as diverticulitis, appendicitis, and pelvic inflammatory disease [1]. Management consists of broad-spectrum antibiotics, with drainage being necessary to achieve source control in approximately 25% of cases [2]. Drainage is also recommended for abscesses greater than 3 cm (cm) in size [3]. Traditionally, source control is achieved via percutaneous drainage by interventional radiology (IR) or surgically. However, the location of pelvic abscesses near the rectum or left colon can make external drainage challenging with an increased risk of anal sphincter injury and potential incontinence. In addition, surgical drainage is associated with significant morbidity and mortality and percutaneous drainage may require multiple interventions and the placement of an indwelling external catheter, leading to increased patient discomfort [4]. In recent years, endoscopic ultrasound (EUS)-guided drainage has emerged as a minimally invasive alternative for the management of complex pelvic abscesses with the advantage of real-time sonographic visualization of the abscess and stenting to facilitate drainage while avoiding potential damage to blood vessels and nerves. Historically, double-pigtail plastic stents were frequently used for EUS-guided drainage [5,6]. However, complications such as stent migration and inadequate drainage requiring additional endoscopic or surgical procedures have been reported [7].

Recently, lumen-apposing metal stents (LAMS) have been increasingly used for a wide range of therapeutic gastrointestinal procedures. They were originally developed for the management of peripancreatic fluid collections and walled-off necrosis and were approved by the United States Food and Drug Administration (FDA) in 2013. Their implementation, while effective, notably came with a steep learning curve; analysis by Facciorusso et al. demonstrated a minimum required 15 procedures before rates of adverse events were at their lowest [8]. In recent years, however, they have demonstrated effectiveness in managing post-surgical fluid collections following exploratory laparotomies, gastric bypasses, and intestinal anastomoses, including those that had previously failed percutaneous or surgical drainage [9,10,11]. While most data are related to their use in peripancreatic fluid collections, the cost-effectiveness of LAMS compared to surgical drainage has proven to be one of its strengths [12]. LAMS utilizes a barbell-shaped design with large flanges on each end which allows for anchorage in two adjacent lumens to prevent stent migration [13]. The inner lumen of the LAMS is larger than that of either plastic or self-expanding metal stents, allowing for more effective drainage and passage of solid necrotic debris. The lumen is also large enough to pass a standard gastroscope through for direct endoscopic debridement if needed [14]. We report the initial experience of EUS-guided LAMS application for the drainage of complex pelvic abscesses at a large safety-net hospital.

## 2. Materials and Methods

### 2.1. Study Population and Data Collection

Participants in this study consisted of patients who underwent EUS-guided drainage of complex pelvic abscesses with cautery-enhanced LAMS (AXIOS, Boston Scientific, Boston, MA, USA) between July 2020 to June 2024 in our institution, a tertiary safety-net hospital. Abscess size and location were confirmed by cross-sectional imaging prior to LAMS placement. All cases underwent multidisciplinary review with surgery and IR prior to LAMS-assisted drainage and were deemed not amendable to drainage by IR. All LAMS were placed under general anesthesia and were performed by two interventional endoscopists (SR and VE) with extensive experience in EUS and LAMS placement for over 10 years. The stents were removed during scheduled outpatient endoscopic follow-up visits after abscess resolution was confirmed via cross-sectional imaging. After drainage, patients were placed on a low-residue diet with osmotic laxatives and instructions to avoid constipation.

Data were retrospectively collected on patient demographics, procedure indications, fluid collection size, stent characteristics, stent dwell time, complications related to LAMS placement, and the need for additional IR-guided or surgical procedures. Time from LAMS placement to discharge and total length of hospitalization was also recorded.

All patients were given standard colonoscopy preparation with an osmotic laxative and a clear liquid diet the day before the procedure. Patients were placed in the left lateral decubitus position. Flexible sigmoidoscopy was performed with an adult endoscope or pediatric colonoscope to approximate the location of fluid collection and evaluate for evidence of extrinsic compression. The linear array therapeutic echoendoscope (Olympus GF-UCT180, Olympus America Inc., Cypress, CA, USA) was then introduced and advanced to the level of the fluid collection. A fluid collection was considered to be adequate for drainage if its size was 4 cm or larger and the distance from the intestinal lumen to the collection was less than 10 mm. The collection was accessed with an electrocautery-enhanced lumen-apposing metal stent without guidewire advancement prior to puncture. LAMS was deployed under EUS and fluoroscopic guidance. Successful deployment was followed by drainage of purulent material into colonic lumen.

Technical success was defined as the successful placement of the LAMS into the pelvic abscess. Clinical success was defined as the resolution of clinical symptoms and radiographic evidence of abscess resolution on cross-sectional imaging.

### 2.2. Statistical Analysis

Descriptive statistics were performed to summarize patient demographics, abscess and stent characteristics, procedure details, and clinical outcomes. Continuous variables were reported as mean (standard deviation) or median (range). Categorical variables were reported as frequencies and percentages.

### 2.3. Ethics

Patient confidentiality and data protection was ensured throughout this study.

## 3. Results

**Figure 1:**
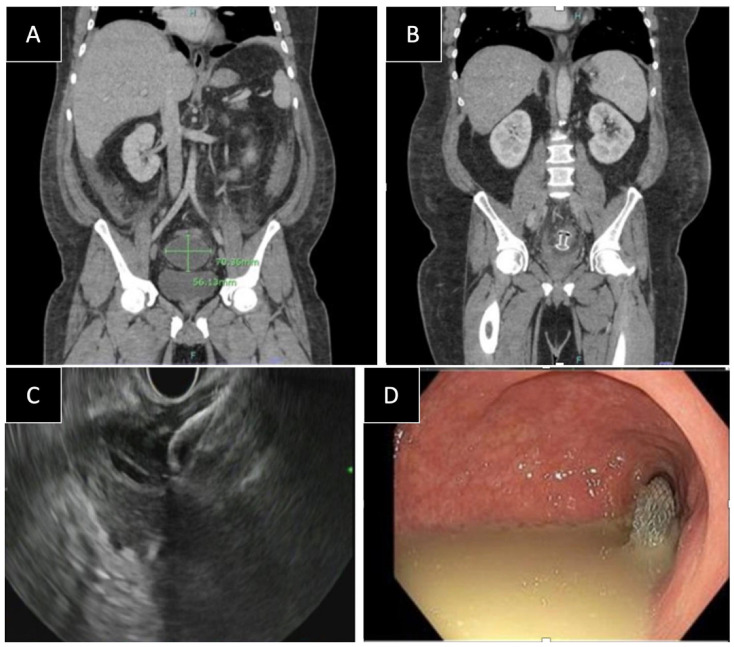
(**A**) 70 mm × 56 mm pelvic fluid collection. (**B**) Abdominal-pelvic CT performed 48 h post-LAMS placement with complete resolution of the pelvic fluid collection. (**C**) Endoscopic ultrasound view of LAMS deployment. (**D**) Endoscopic view post-LAMS deployment.

Eleven patients underwent EUS-guided drainage of complex pelvic abscesses with cautery-enhanced LAMS during the study period (Figure 1; Table 1 and Table 2). The mean age was 39 years old and 82% of patients were male. Complicated diverticulitis was the most common etiology of abscess formation 6/11 (55%), followed by gunshot injury, perforated appendicitis, and infection of a presacral cyst. The average time from presentation to drainage was 7 days (1–18 days). The average abscess size was 7.2 cm (3.9–12.0 cm). The most common LAMS size deployed was 15 mm × 10 mm (63.6%), followed by 10 mm × 10 mm (36.4%). The average distance from the site of LAMS placement to the anal verge was 17.0 cm (5–30 cm). Six of eleven stents (55%) were placed through the left colon and 5/11 (45%) through the rectum. The average time from LAMS placement to discharge was 4 days (1 to 18 days). The average hospital length of stay (LOS) was 10.5 days (2 to 24 days).

All LAMS placements were technically and clinically successful. Two patients required the placement of a coaxial plastic double-pigtail stent through the LAMS to achieve adequate drainage due to a significant amount of solid debris within the collection. All patients had resolution of symptoms and pelvic abscesses after LAMS placement confirmed by cross-sectional imaging; the median stent dwell time was 28 days (17–42 days). The average duration of follow-up after LAMS insertion was 25 weeks (SD 35.6 weeks); the median follow-up time was 8.4 weeks (0.4–118.6). Fistula closure was not routinely performed following LAMS removal given the fistulas were likely to spontaneously close. No recurrence of pelvic abscesses was noted during the follow-up period.

There were no LAMS-related complications such as bleeding, perforation, or stent migration. No patients required additional IR or surgical procedures after LAMS removal. One patient reported post-procedural pelvic pain, although chronic pelvic pain was present prior to LAMS placement. Another patient reported self-limited post-procedural nausea and right lower quadrant pain.

One case of a complex pre-sacral cyst that was transformed into an abscess after failed transvaginal fenestration was successfully drained with LAMS. Prior to LAMS drainage, the patient had multiple admissions for recurrent pelvic abscess due to cyst re-infection. The patient required a prolonged LAMS dwell time in an attempt to create a permanent fistulous tract to prevent cyst re-infection. The patient remained asymptomatic for greater than 6 months after LAMS removal. A patient with an abscess secondary to a gunshot wound had a prolonged LAMS dwell time due to delays over adequate colonic preparation prior to removal. The patient tolerated this prolonged period well and the stent was successfully removed; the patient remained asymptomatic at their 1-month follow-up. Lastly, one patient with an abscess secondary to diverticulitis declined LAMS removal; cross-sectional imaging 48 h after LAMS placement showed near-complete resolution of the fluid collection and the patient remained asymptomatic at day 45. The patient was eventually lost to follow-up. 

## 4. Discussion

EUS-guided LAMS placement led to the complete resolution of the abscess in all cases in our cohort of patients. The procedure itself was well-tolerated without complications. There were no episodes of proximal or distal stent migration. Patients were able to be discharged relatively quickly after LAMS placement (median of 3 days) with three patients safely discharged on post-operative day one. Though larger LAMS sizes (15 mm) were selected to ensure adequate drainage, none failed to achieve resolution of the fluid collection. The median overall length of hospitalization was 7 days. Our findings suggest that LAMS can serve as a safe and effective alternative to conventional drainage techniques with high success rates and low rates of complications.

In our study, LAMS were deployed in a wide variety of clinical scenarios. Abscess size ranged from 3.9 cm to 12.0 cm and LAMS were placed anywhere between 5 cm to 30 cm from the anal verge. There were six transcolonic and five transrectal placements. While transrectal drainage has been previously reported in the surgical literature, data on transcolonic drainage is relatively limited given the recent advances in the EUS-guided approach. In the studies examining EUS-guided drainage, transcolonic approaches were much less frequently reported with a meta-analysis from 2021 describing 83.7% perirectal approaches and 16.7% pericolonic approaches [5]. Because of the colon’s proximal location to the peritoneal reflection, there was concern that the colon’s thinner wall could precipitate a higher risk of perforation with transcolonic drainage [15]. However, subsequent studies found similar rates of complications between transrectal and transcolonic approaches using pigtail stents [16]. In our experience, the safety profile of both transcolonic and transrectal LAMS placements were similar without perforation or excess bleeding. Of our six patients who underwent transcolonic drainage, there were no major adverse events with only one patient reporting self-limited post-procedural symptoms.

A meta-analysis on outcomes of EUS-guided drainage of pelvic abscesses, mostly with double pigtail stents, reported an adverse event rate of 9.4%, which is comparable to that of IR-guided drainage which ranges from 5.6 to 10.0% [5]. One case of perforation was reported in the meta-analysis, which occurred in a patient with a diverticular abscess drained via transcolonic LAMS. The patient reported post-procedural abdominal pain and displayed clinical signs of sepsis on post-operative day one and required surgical intervention which revealed a perforation of the diverticulum. The authors reported a favorable post-operative outcome [17]. There were no instances of perforation or other adverse events in our cohort.

LAMS have been increasingly used in the management of post-surgical fluid collections. In a retrospective analysis by Chhabra et al., five patients with post-surgical pancreatic fluid collections achieved both technical and clinical success via LAMS. Two patients from this cohort had failed previously attempted percutaneous drainage [18]. Similarly, in a case of a post-Whipple abscess presented by Kumar et al., successful drainage was achieved by transjejunal placement of LAMS [19]. Lastly, in a multicenter analysis conducted by Mudireddy et al., 47 patients with such collections (55% due to postoperative pancreatic duct leaks) underwent EUS-guided LAMS-assisted drainage, with a majority of patients receiving transgastric placement [9]. Though a majority of these studies evaluated the use of LAMS in pancreatic fluid collection management, they most importantly demonstrate the post-operative success of LAMS throughout the GI tract. In our cohort, three patients with post-operative fluid collections were successfully drained with LAMS (one secondary to an anastomotic colonic leak and two due to operative management of a gunshot injury). Ultimately, these studies demonstrate the growing recognition of LAMS’ diverse applications, not only in the anatomical sense but also in the etiologies of fluid collections they can manage.

Prior studies have reported a wide range of stent dwell times needed to achieve adequate drainage. Meylemans et al. compared EUS-guided drainage via biliary drainage catheters with surgical transrectal drainage of pelvic abscesses in a cohort of 46 patients [20]. The EUS group had a mean stent dwell time of 42 days, compared to 13 days in the surgical group. This was likely due to logistic reasons as patients in the surgical group required their drain to be removed prior to discharge, while patients in the EUS group were discharged with their stent in place with plans for removal during outpatient follow-up. In our experience with LAMS, the mean stent dwell time of 28 days was shorter than that of the EUS-guided biliary drainage catheter group but longer than that of the surgical group in Meylemans’ study. Like the EUS group, our patients were discharged with the LAMS in place. The stents were removed during scheduled outpatient follow-up visits after abscess resolution was confirmed via cross-sectional imaging. Of note, it is possible that stent dwell time may have been confounded by logistical difficulties in scheduling outpatient follow-up. Regardless, the flexibility to discharge patients with LAMS in place may be an important factor in selecting this therapeutic approach over surgical management. Additionally, though there is discussion over the association between bleeding risk and stent dwell times in peripancreatic fluid collections, there is limited data on risk for pelvic abscesses. In an analysis of 516 patients with peripancreatic fluid collections who received LAMS, 5.6% experienced bleeding [21]. In an attempt to minimize this risk, we aimed to remove stents within 3 to 4 weeks as per manufacturer recommendations for peripancreatic fluid collections. Nevertheless, future prospective studies are needed to determine the optimal stent dwell time to allow for appropriate outpatient follow-up intervals while reducing adverse outcomes.

Similar to other EUS-guided drainage modalities, LAMS has the potential to decrease hospital LOS compared to surgical or IR-guided percutaneous drainage. Early data demonstrated the potential for LAMS to achieve greater outcomes than percutaneous intervention, as the presence of loculations or complex pelvic anatomy may interfere [22]. Additionally, with percutaneous drainage, if the drain is not removed prior to discharge, patients may require home nursing care or education to care for their drain at home. This may influence clinicians to keep their patients hospitalized until drainage is complete, leading to shorter stent dwell times and longer LOS. In a retrospective study of 39 patients who underwent percutaneous transgluteal drainage of pelvic abscesses by IR, the mean duration of drainage was 8.3 days, which was longer than the mean post-procedural LOS of 4.2 days in our study. The percutaneous approach also required diligent catheter care to maintain catheter patency, which included irrigation of the indwelling catheter every 6 to 8 h [23]. In a larger study of 126 patients who underwent CT-guided percutaneous drainage of deep pelvic abscesses, authors reported a similar mean duration of drainage of 8 days [24]. The overall LOS was not reported in either study. Of note, in Meylemans’ study, there was no significant difference in overall LOS between the EUS group and the surgical group (24 days vs. 20 days, *p* = 0.56) [20]. Differences in LOS between EUS-guided LAMS versus EUS-guided pigtail catheter drainage have not been established but are likely small given their procedural similarities. Varadajulu et al. studied outcomes of EUS-guided drainage of pelvic abscesses via pigtail stents in a cohort of 25 patients [12]. The overall LOS was not reported but the median post-procedural LOS was 2 days. Our study showed similar findings with a median post-procedural LOS of 3 days and a median overall LOS of 8 days.

A prior study using double pigtail stents noted that the success rate for EUS-guided drainage of pelvic abscesses due to complicated diverticulitis was lower than that of abscesses due to other causes (25% vs. 97%, *p* = 0.002) [14]. In this retrospective study, EUS-guided fine needle aspiration (FNA) was performed, followed by guidewire insertion and sequential dilation of the tract using an ERCP cannula and biliary balloon dilators. A double-pigtail stent was then placed to facilitate drainage. The authors attributed the low drainage success rates of diverticular abscesses to the multiloculated nature of the diverticular fluid collections and the thick viscosity of its fluid contents. Most of the pelvic abscesses in our study were due to diverticulitis and all were drained successfully. The large diameter of LAMS likely facilitates drainage and has the advantage of requiring fewer steps and dilations, lowering the risk of perforation. Though the authors concluded that diverticular abscesses with poorly defined walls, debris, and difficulty aspirating abscess contents with a 19-gauge needle were best managed surgically, these abscesses that were once deemed non-amendable to endoscopic drainage can likely be treated using EUS-guided LAMS placement. In our cohort, coaxial stents were used with LAMS if an abscess contained a significant amount of solid debris to optimize drainage and decrease risk of LAMS occlusion.

Though not the primary goal of our study, the impact of drainage modality on patients’ quality of life should be considered. Transgluteal, transrectal, transvaginal, and percutaneous approaches, while ostensibly less invasive than surgical intervention, may not sufficiently minimize the impact on patients post-procedurally. Several publications have weighed in on the impact of LAMS versus standard of care percutaneous drains (PD); the authors cite infection risk, risk of percutaneous fistula, the need for drain repositioning or flushing, among other concerns, as integral to the implementation of LAMS [25,26]. In one such case, a patient presenting with a rectouterine pouch abscess was considered for placement of a transgluteal, 14Fr catheter, but was adequately managed with a LAMS that achieved near-complete resolution of the abscess within 5 days [21]. Several of our patients demonstrated an ability to tolerate prolonged LAMS stenting with minimal impact in the outpatient setting. Of the eleven reported here, 36% (4/11) tolerated the stent for nearly a month until resolution of the abscess necessitated its removal. One patient, who was ultimately lost to follow-up, remained asymptomatic despite indwelling stent by day 45. While, ideally, stent dwell time is minimized to avoid complications, therapies should account for the potential need for a prolonged clinical course. Ultimately, individual patient factors will dictate the selected therapeutic approach, but we demonstrate the potential for LAMS to minimize impact on patients’ quality of life and remain tolerable for extended periods.

There are several limitations to our study. Mainly, this is a retrospective analysis from a single academic safety-net center, which could have affected the generalizability of the results. The number of patients evaluated, albeit small, is comparable to those previously reported [5]. The logistic challenges with scheduling outpatient follow-up appointments in our underserved patient population may have influenced the stent dwell times in our cohort. The lack of a control group limits the ability to directly compare the outcomes of LAMS placement with alternative interventions such as double-pigtail plastic stents alone, surgical approaches, or percutaneous drainage. Thus, the superiority or equivalence of LAMS cannot definitively be determined.

In our experience, EUS-guided LAMS placement is a promising minimally invasive therapeutic approach to the drainage of complex pelvic abscesses. It should especially be considered in patients who are not appropriate candidates for percutaneous or surgical approaches and ultimately could become a primary treatment modality. The anticipated clinical course of patients, specifically those who require extended periods of drainage, could also be considered given tolerability of LAMS as noted in our experience. Our study highlights the high technical and clinical success, and low complication rates of LAMS drainage. Future studies are needed to determine the optimal procedure protocol and stent dwell times and to establish LAMS as the standard of care for the management of complex pelvic abscesses.

## Figures and Tables

**Table 1:** Patient and procedure characteristics.

Age	Sex	Etiology of Abscess	Abscess Size (cm)	LAMS Size (mm)	LAMS Distance from Anal Verge (cm)	Stent Dwell Time (Days)	Presentation to LAMS Placement (Days)	LAMS Placement to Discharge (Days)	Length of Hospitalization (Days)	Resolution Achieved on Imaging	Post-Procedure Adverse Events
25	M	Gunshot injury	6.0 × 3.0	15 × 10	20	23	13	3	16	Yes	none
25	M	Diverticulitis	8.8 × 5.5	10 × 10	25	26	5	2	7	Yes	none
48	M	Diverticulitis	12.0 × 9.5	15 × 10	25	Lost to follow up	2	3	5	Yes *	none
60	M	Diverticulitis	6.0 × 4.0	15 × 10	20	17	6	18	24	Yes	none
61	M	Diverticulitis	12.2 × 2.6	15 × 10 + Pigtail stent	25	27	17	7	24	Yes	none
46	F	Infected presacral cyst	8.3 × 5.2	15 × 10 + Pigtail stent	6	28	N/A	2	3	Yes	pelvic pain **
12	M	Perforated appendix	3.9 × 1.1	10 × 10	30	19	14	4	18	Yes	self-limiting nausea, RLQ pain
40	M	Diverticulitis	4.8 × 2.4	15 × 10	6	45	7	1	8	Yes	none
30	F	Diverticulitis	6.5 × 3.5	10 × 10	15	25	2	4	6	Yes	none
62	M	Post-surgical anastomotic leak	4.5 × 3.7	10 × 10	5	29	1	1	2	Yes	none
19	M	Gunshot injury	8.0 × 6.0	15 × 10	7	42	2	1	3	Yes	none

* Based on CT scan results 48 h after LAMS placement and resolution of symptoms on day 45 after placement. ** Pelvic pain was present prior to LAMS placement.

**Table 2:** Summary of patient demographics, abscess characteristics, and procedure outcomes.

Age, years (mean, SD)	38.9 (17.8)
Sex	
Male	9 (81.8%)
Female	2 (18.2%)
Etiology of abscess	
Diverticulitis	6 (54.5%)
Gunshot injury	2 (18.2%)
Infected presacral cyst	1 (9.1%)
Perforated appendix	1 (9.1%)
Post-surgical fistula	1 (9.1%)
Abscess size	
Mean (SD)	7.2 (2.8)
Median (range)	6.0 (3.9, 12.0)
LAMS size	
15 × 10 mm	7 (63.6%)
10 × 10 mm	4 (36.4%)
LAMS distance from anal verge, cm	
Mean (SD)	17.0 (9.0)
Median (range)	20.0 (5, 30)
Colonic placements	6 (54.5%)
Rectal placements	5 (45.5%)
Stent dwell time, days	
Mean (SD)	28.1 (9.0)
Median (range)	26.5 (17, 42)
Presentation to LAMS placement, days	
Mean (SD)	6.9 (5.8)
Median (range)	5.5 (1, 17)
LAMS placement to discharge, days	
Mean (SD)	4.2 (4.9)
Median (range)	3.0 (1, 18)
Length of hospitalization	
Mean (SD)	10.5 (8.4)
Median (range)	7.0 (2, 24)
Time until follow-up, weeks	
Mean (SD)	25.0 (35.6)
Median (range)	8.4 (0.4, 118.6)

## Data Availability

Data available on request due to restrictions (e.g., privacy, legal or ethical reasons).
